# Supplementary material for: Simultaneous loss of interlayer coherence and long-range magnetism in quasi-two-dimensional PdCrO2
Source: Nat Commun. 2017 Apr 13;8:15001. doi: 10.1038/ncomms15001 (PMC5399288; doi:10.1038/ncomms15001)
Supplement: Supplementary Information — Supplementary Figures, Supplementary Notes and Supplementary References [file ncomms15001-s1.pdf]

SUPPLEMENTARY NOTE 1. ANGLE-DEPENDENT MAGNETORESISTANCE

A previous interlayer magnetoresistance study in $PdCoO_2$ by Takatsu *et al.* [1] showed a striking angle dependence upon azimuthal rotation of the magnetic field within the conducting plane that was attributed to high mobility of the conduction electrons and fine details of the hexagonal Fermi surface of $PdCrO_2$. We note however that the data reported in the Takatsu paper were taken with the magnetic field aligned approximately 3° away from the conducting plane. As shown in Figure 2**d** of the main manuscript, the Hanasaki peak is suppressed completely once the field is rotated 2° away from the conducting plane. Thus, the sharp peaks in the interlayer magnetoresistance reported in Ref. [1] are not associated with the Hanasaki peaks discussed in the main paper.

Additionally, it should be pointed out that while the polar ADMR are extremely sensitive to details of the Fermi surface topology—as can be seen by inspection of the polar ADMR curves for $PdCrO_2$ (Figure 2**b** of the main manuscript) and $PdCoO_2$ (Figure 5**a** of [2])—the Hanasaki peak itself is similar in form in both systems. Indeed, to be visible, the Hanasaki peak requires only the existence of a three-dimensional Fermi surface (provided that $\omega_c\tau$ is large enough) and its width is determined uniquely by the ratio between k_F and $t_\perp$. It is therefore only weakly dependent on other details of the band structure.

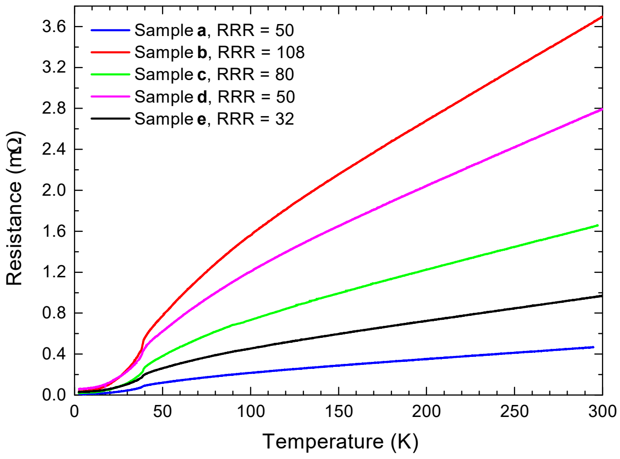

Supplementary Figure 1. | **The** c**-axis resistance of different samples**. The highest quality sample, with residual resistivity ratio of 108, was chosen for our angular-dependence measurements.

SUPPLEMENTARY NOTE 2. BACKGROUND SUBTRACTION

For analysis of the coherence peak sharpness, the resistivity values were normalized to a common scale as given by

$$\rho_{\rm N}(T,\theta,H)=\frac{\rho_{\rm c}(T,\theta,H)-\rho_{\rm c}(T,\theta=0°,H)}{\rho_{\rm c}(T,\theta=0°,H=0\,{\rm T})}. \quad (1)$$

The broad quasi-sinusoidal background was then removed by subtracting the $\rho_{\rm N}(T,\theta,H)$ curve at 44 K, which has no AMRO nor coherence peak, via

$$\rho_{\rm N.B.}(T,\theta,H)=\rho_{\rm N}(T,\theta,H)-\alpha\rho_{\rm N}(44\,{\rm K},\theta,H), \quad (2)$$

where the multiplicative factor α broadly corrects for the change in $\omega_{\rm c}\tau$ with temperature and 'N.B.' stands for 'no background'. The factor α was found by empirically scaling the 44 K data until $\rho_N(44\,{\rm K},\theta,H)$ matched $\rho_N(T,\theta,H)$ in the featureless low-angle region (0–30°). This results in a $\rho_{\rm N.B.}(T,\theta,H)$ that is approximately zero at low angles, with a series of peaks at higher angles. Thus one has a complete removal of the quasi-sinusoidal background, leaving only the AMRO features and the Hanasaki coherence peak that are observed at higher angles. As an example, see Supplementary Figure 3.

Using the 44 K curve to subtract the background in this way does not affect the analysis of the sharpness of the coherence peak. This is shown in the plot of $\mathrm{d}^2\rho/\,\mathrm{d}\theta^2$ in Supplementary Figure 3**b** of the main manuscript, where $\mathrm{d}^2\rho/\,\mathrm{d}\theta^2$ at $\theta=90°$ is essentially zero for all four data points above 37.5 K. This confirms that the 44 K curve does not have a coherence peak, and the implies successful subtraction of the quasi-sinusoidal background in a way that does not influence our analysis of the temperature dependence of the Hanasaki peak.

SUPPLEMENTARY NOTE 3. ESTIMATE OF $\hbar/\tau$ AND $\omega_c\tau$

The mean free path can be estimated from the Drude formulae for a simple cylindrical Fermi surface:

$$\tau=\frac{m^*}{ne^2\rho_{ab}},\quad n=\frac{k_{\rm f}^2}{2\pi d} \quad (3)$$

where n is electronic density, m^* is the effective mass, $d=6.03\mathring{A}$ is the interplanar distance, e is the electron charge and $k_{\rm f}$ is the Fermi wave-vector.

Thus given that $\rho_{ab}(37.5\,{\rm K})=0.7(1)\,\mu\Omega\,{\rm cm}$ [3, 4], and assuming that the electronic transport is dominated by the biggest non-breakdown orbit (γ) for which $k_{\rm f}=0.57(3)\,\mathring{\rm A}^{-1}$ and $m^*=1.37(2)m_{\rm e}$, we obtain $\hbar/\tau$ of 0.8(1) meV at $T_{\rm N}$, and $\omega_c\tau=eB\tau/m^*$ of 3.0(3) at 30 T and 37.5 K.

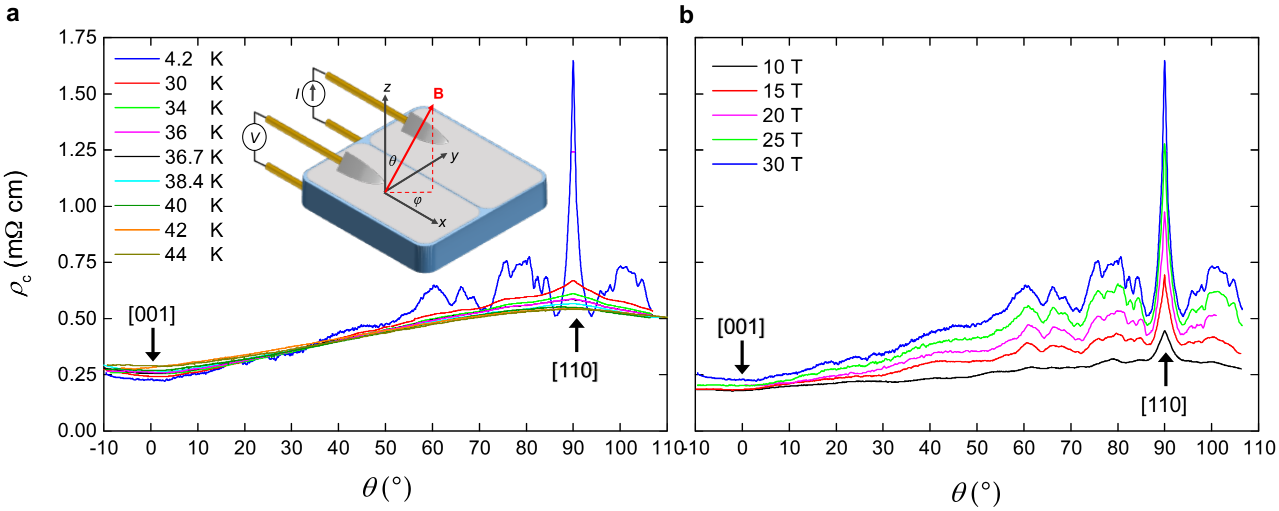

Supplementary Figure 2. | **Angle-dependent magnetoresistance**. Evolution of the c-axis resistivity $\rho_c(\theta)$ as the sample is rotated around the polar axis, from the magnetic field normal (0°) to parallel (90°) to the crystal planes. Inset: schematic diagram of the four-contact setup used to measure c-axis resistivity, showing the definition of the polar (θ) and azimuthal (ϕ) angles. **a**, Measurements at a range of temperatures below and above the long-range ordering temperature $T_N = 37.5\,K$, at a fixed field of 30 T. **b**, Measurements at fixed fields of 10–30 T in 5 T increments, at a fixed temperature of 4.2 K. The direction of the applied field at 0° and 90° are indicated.

Finally, it should be noted that in contrast to quantum oscillations measured by the de Haas-van Alphen effect, where the amplitude of $\omega_c\tau$ can be suppressed by both small- and large-angle scattering events, angle-dependent magnetoresistance (ADMR), being a transport property, is not affected or degraded by small-angle scattering. This is best illustrated in Ref. [5] where the polar ADMR in the interplane magnetoresistance of an overdoped cuprate could be fitted by precisely the same $\omega_c\tau$ value that is obtained from in-plane Hall effect measurements. Thus, the $\omega_c\tau$ product obtained from the interlayer magnetoresistance is, in principle, identical to that estimated from zero-field resistivity measurements.

SUPPLEMENTARY NOTE 4. FIELD-DEPENDENCE OF THE HANASAKI PEAK

It is interesting to investigate the field-dependence of the amplitude of the Hanasaki peak since any strong variation in the scattering rate with field, e.g. due to a change in the spin fluctuation spectrum, could be reflected in a departure from the expected quadratic dependence of the peak amplitude with field. In this section, we thus compare the field-dependence of the Hanasaki peak in $PdCrO_2$ with that measured in non-magnetic $PdCoO_2$. The corresponding amplitudes are plotted as a function of field in Supplementary Figure 4. We find that the field-dependence in both systems is quadratic in field to within our experimental uncertainty. This finding suggests that the antiferromagnetic fluctuation rate does not vary appreciably at least within the field range of our experiments. With regards the cuprates, where antiferromagnetic fluctuations are clearly present, their influence on the magnetotransport, vis-á-vis the field-dependence of the magnetoresistance, for example, is also found to be negligible (see, *e.g.* Ref. [6]) despite the fact that it may have a significant impact on its temperature dependence [6].

SUPPLEMENTARY REFERENCES

[1] Takatsu, H. *et al.* Extremely large magnetoresistance in the nonmagnetic metal $PdCoO_2$. *Phys. Rev. Lett.* **111**, 056601 (2013).

[2] Kikugawa, N. *et al.* Interplanar coupling-dependent magnetoresistivity in high-purity layered metals. *Nat Commun* **7** (2016).

[3] Hicks, C. W. *et al.* Quantum oscillations and magnetic reconstruction in the delafossite $PdCrO_2$. *Phys. Rev. B* **92**, 014425 (2015).

[4] Takatsu, H., Yonezawa, S., Michioka, C., Yoshimura, K. & Maeno, Y. Anisotropy in the magnetization and resistivity of the metallic triangular-lattice magnet $PdCrO_2$. *J. Phys. Conf. Ser.* **200**, 012198 (2010).

[5] Hussey, N. E., Abdel-Jawad, M., Carrington, A., Mackenzie, A. P. & Balicas, L. A coherent three-dimensional fermi surface in a high-transition-temperature superconductor. *Nature* **425**, 814–817 (2003).

[6] Harris, J. M. *et al.* Violation of kohler's rule in the normal-state magnetoresistance of $YBa_2Cu_3O_{7-\delta}$ and $La_2Sr_xCuO_4$. *Phys. Rev. Lett.* **75**, 1391–1394 (1995).

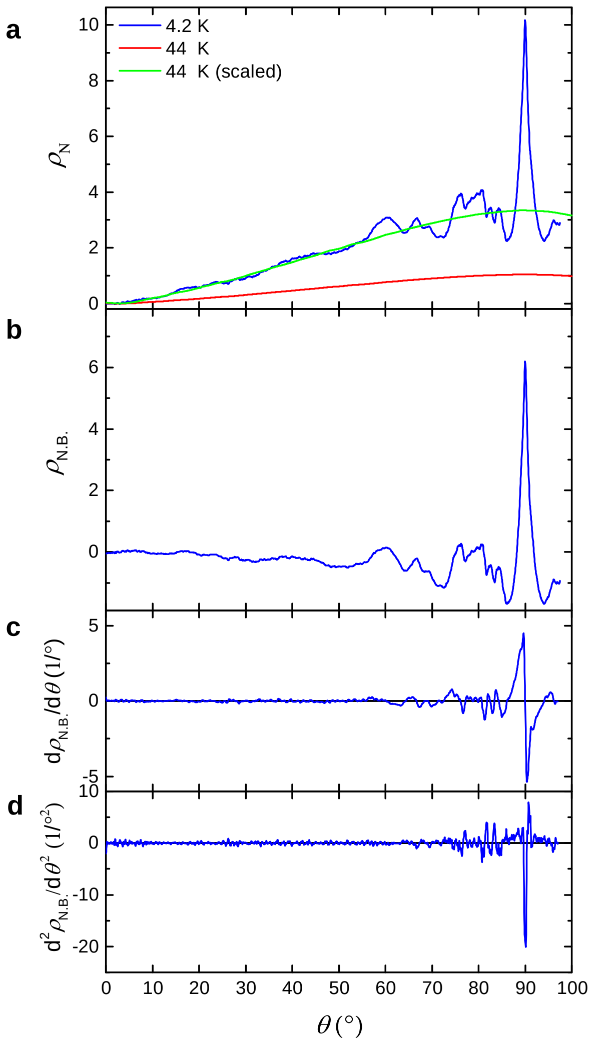

Supplementary Figure 3. | **Background subtraction.** **a**, The normalized resistivity values (as defined in Supplementary Equation 1) for measurements at 4.2 K and 44 K, at a fixed field of 30 T. The green line is a scaled copy of the 44 K data, as described in the main text. **b**, The result after subtraction of the scaled scaled 44 K data from the 4.2 K curve. **c** and **d** are the first and second differentials, respectively, of the curve shown in **b**.

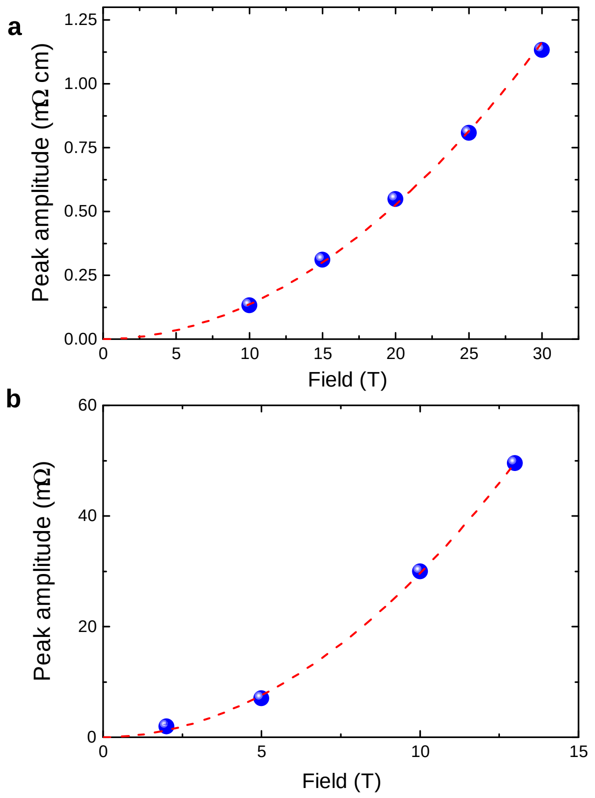

Supplementary Figure 4. | **Field dependence of the coherence peak.** The evolution of the amplitude of the Hanasaki coherence peak (as measured from base to peak) for **a**, $PdCrO_2$ at 4.2 K and **b**, $PdCoO_2$ at 1.6 K. The red lines are fit to the the power law $a + bH^n$. For both compounds the exponent n is found to be 2.0.
